# Robustness of Spike Deconvolution for Neuronal Calcium Imaging

**DOI:** 10.1523/JNEUROSCI.3339-17.2018

**Published:** 2018-09-12

**Authors:** Marius Pachitariu, Carsen Stringer, Kenneth D. Harris

**Affiliations:** ^1^Howard Hughes Medical Institute, Janelia Research Campus, Ashburn, Virginia 20147,; ^2^University College London, Institute of Neurology, London WC1N 3BG, United Kingdom,; ^3^University College London, Department of Neuroscience, Physiology, and Pharmacology, London WC1E 6BT, United Kingdom, and; ^4^Gatsby Computational Neuroscience Unit, London W1T 4JG, United Kingdom

**Keywords:** calcium imaging, spike deconvolution, spike inference

## Abstract

Calcium imaging is a powerful method to record the activity of neural populations in many species, but inferring spike times from calcium signals is a challenging problem. We compared multiple approaches using multiple datasets with ground truth electrophysiology and found that simple non-negative deconvolution (NND) outperformed all other algorithms on out-of-sample test data. We introduce a novel benchmark applicable to recordings without electrophysiological ground truth, based on the correlation of responses to two stimulus repeats, and used this to show that unconstrained NND also outperformed the other algorithms when run on “zoomed out” datasets of ∼10,000 cell recordings from the visual cortex of mice of either sex. Finally, we show that NND-based methods match the performance of a supervised method based on convolutional neural networks while avoiding some of the biases of such methods, and at much faster running times. We therefore recommend that spikes be inferred from calcium traces using simple NND because of its simplicity, efficiency, and accuracy.

**SIGNIFICANCE STATEMENT** The experimental method that currently allows for recordings of the largest numbers of cells simultaneously is two-photon calcium imaging. However, use of this powerful method requires that neuronal firing times be inferred correctly from the large resulting datasets. Previous studies have claimed that complex supervised learning algorithms outperform simple deconvolution methods at this task. Unfortunately, these studies suffered from several problems and biases. When we repeated the analysis, using the same data and correcting these problems, we found that simpler spike inference methods perform better. Even more importantly, we found that supervised learning methods can introduce artifactual structure into spike trains, which can in turn lead to erroneous scientific conclusions. Of the algorithms we evaluated, we found that an extremely simple method performed best in all circumstances tested, was much faster to run, and was insensitive to parameter choices, making incorrect scientific conclusions much less likely.

## Introduction

Two-photon calcium imaging can be used to monitor the activity of populations of up to 10,000 neurons (Pachitariu et al., 2016; Stringer et al., 2018). Nevertheless, calcium-sensitive fluorescence signals are an indirect readout of cellular activity. Therefore, accurate and well-calibrated data processing methods will be required to make optimal use of this activity (Vogelstein et al., 2010; Andilla and Hamprecht, 2014; Reynolds et al., 2015; Deneux et al., 2016; Theis et al., 2016; Friedrich et al., 2017; Jewell and Witten, 2017; Sebastian et al., 2017; Kazemipour et al., 2018). One important problem is developing methods for spike detection: inferring the times of action potentials from the fluorescence traces. The earliest such methods rely on spike deconvolution algorithms, which infer a spike train under the assumption that the fluorescence trace represents an approximate convolution of the underlying spike train with the cell's calcium response (Vogelstein et al., 2010). This is often a good approximation (Chen et al., 2013), although situations exist when it breaks down. More complex spike deconvolution algorithms take these extreme cases into account (Deneux et al., 2016).

Recently, a new approach to spike detection, based on supervised learning, has been claimed to outperform several existing deconvolution algorithms (Theis et al., 2016). Supervised algorithms learn to solve the spike detection problem by training on “ground truth” data where spike times are also measured electrophysiologically. In principle, such methods should give the most accurate results: however, they may generalize poorly to “out-of-sample” data (i.e., recordings made under different conditions to the available training data).

Since the supervised approach was first introduced, ground truth data were released in a public competition called “spikefinder” (Berens et al., 2018), which has allowed the comparison of several old and new algorithms. Three of the better performing algorithms (“Elephant,” “Purgatorio,” and “convi6”) use supervised methods based on convolutional neural networks (CNNs) and appear to slightly outperform unsupervised methods based on non-negative deconvolution (NND; “oopsi”, “Suite2p”). However, this performance may be due to specific design features of the spikefinder challenge, rather than true improvements in spike deconvolution quality. First, the spikefinder benchmarks are run on in-sample data and may thus not reflect generalization performance to new recordings. Second, multiple metrics for the similarity of decoded and actual spike trains are possible; because supervised methods can be trained to optimize the particular metric used, they will have an advantage over unsupervised methods, unless the latter are also optimized for the particular quality metric used.

We show here that NND, with very simple parameter settings, and using the fast OASIS implementation (Friedrich et al., 2017), outperforms supervised algorithms, when it is: (1) evaluated on out-of-sample data, and (2) adapted to the performance metric of the spikefinder challenge. In addition, we find that NND is highly robust to assumptions on the assumed shape of the calcium response to single spikes (henceforth called a “kernel”), such that a simple decaying exponential kernel performs better than more biologically accurate kernels that include a rising time segment, and even performs better than kernels estimated directly from ground truth data. Moreover, large changes to the timescale of the exponential kernels did not affect performance significantly, and optimizing these timescales for each cell actually hurts performance. Finally, we propose a new benchmark that can be used without electrophysiological ground truth, and show that simple NND again outperforms other algorithms on this benchmark. The new benchmark can be used to compare algorithms on a wide range of realistic *in vivo* datasets, and we provide a code repository to facilitate these comparisons, accompanied by a release of the large-scale test datasets used here (Pachitariu et al., 2018) (code: https://github.com/MouseLand/pachitariu-et-al-2018a).

## Materials and Methods

### 

#### 

##### Imaging in visual cortex.

All experimental procedures were conducted according to the United Kingdom Animals Scientific Procedures Act (1986). Experiments were performed at University College London under personal and project licenses released by the Home Office following appropriate ethics review.

The experimental methods were similar to those described previously (Dipoppa et al., 2018). Briefly, surgeries were performed in adult mice of either sex (P35-P125) in a stereotaxic frame and under isoflurane anesthesia (5% for induction, 0.5%–1% during the surgery). During the surgery, we implanted a headplate for later head fixation, made a craniotomy with a cranial window implant for optical access, and, on relevant experiments, performed injections of the GCaMP6m virus with a beveled micropipette using a Nanoject II injector (Drummond Scientific) attached to a stereotaxic micromanipulator. Viruses were acquired from University of Pennsylvania Viral Vector Core. Injections of 50–200 nl virus (1–3 × 10^12^ GC/ml) were targeted to monocular V1, 2.1–3.3 mm laterally and 3.5–4.0 mm posteriorly from bregma and at a depth of L2/3 (200–400 μm). Some mice were transgenic and expressed tdtomato in certain cell classes. However, we did not use that information here.

##### NND model.

NND models infer the most likely spike train **s**(*t*), given the fluorescence time course **F**(*t*) and a response kernel **k**. Models based on deconvolution define a cost function of the following form:

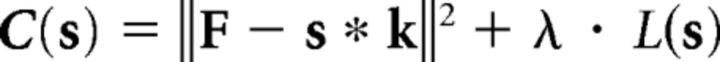
 such that ***s***(*t*) ≥ 0, for all *t*.

Here, s * k describes a temporal convolution of a positive time course s and the kernel k, and *L*(**s**) describes a penalty function on the inferred spike trains.

We tested a suite of three unsupervised spike detection methods. The first is an approximate optimization algorithm where *L*(**s**) = ‖**s**‖_0_ is the L0 norm, that is, the number of nonzero entries in **s** (code available at www.github.com/cortex-lab/Suite2P). The L0 penalty enforces the constraint that the inferred spike trains should be very sparse because neurons fire rarely. The second method has *L*(**s**) = ‖**s**‖_1_, the L1 norm, and chooses the kernel from a parametrized class of functions (Friedrich et al., 2017). The third unsupervised model is unconstrained NND, with *L*(**s**) = 0. For our initial analysis, we chose the sparsity penalty λ for both the L0 and L1 methods in such a way as to output spike trains with similar levels of sparsity: only ∼5% of the deconvolved samples were nonzero, for data sampled at 100 Hz. We then varied the sparsity penalties, as well as the parameters that define the kernel **k**, to understand how they influence performance.

##### Supervised learning models.

We compared performance of these NND algorithms against two supervised methods: the method of Theis et al. (2016) and also a publicly available CNN algorithm (code from https://github.com/PTRRupprecht/Spikefinder-Elephant/tree/master/elephant). In both cases, we used default parameter settings.

##### L0-based spike deconvolution.

We also tested an additional, novel L0 deconvolution algorithm, obtained by developing a novel optimization procedure for a standard spike generation model. This optimization is an extension of a well-known algorithm called “matching pursuit” (Mallat and Zhang, 1993; Smith and Lewicki, 2006), which can be fast enough to apply to the large datasets considered here (Krstulovic and Gribonval, 2006). Briefly, the matching pursuit algorithm identifies putative spikes by their similarity to the calcium kernel. It then subtracts the kernel scaled with an appropriate factor (the “spike amplitude”) from the location of the identified spikes. On successive iterations, more putative spike locations are identified greedily, and their activity subtracted off. This continues until no new spikes can be introduced because they do not have large enough amplitudes to explain a significant portion of the variance.

This basic matching pursuit algorithm is limited by local minima because the greedy procedure cannot always resolve nearby spikes that have overlapping calcium activity. There have been various approaches proposed to address this problem, for example, orthogonal matching pursuit, which reestimates the magnitudes of the atoms in the support set at each iteration (Pati et al., 1993). An “atom” is defined as any potential element that can be picked up by matching pursuit, in our case potential spike location, whereas the “support set” refers specifically to the atoms that have been picked up to some iteration. One disadvantage of orthogonal matching pursuit (OMP) and similar approaches (Soussen et al., 2013) is that they do not allow atoms in the support set to be removed during the optimization. Other recent generalizations do, for example, OMP with replacement or compressive sampling matching pursuit (CoSaMP) (Needell and Tropp, 2009; Jain et al., 2011).

Nevertheless, these generalizations are often computationally intensive and do not explicitly address the problem we encountered most often: early on during greedy extraction, an atom is introduced to account for an entire burst of spikes, and is thus introduced at the average time of the spikes in the burst. To avoid local optima while replacing this single atom with two smaller ones would require adjusting the relative timing of the original atom once others are introduced nearby. However, such a step requires first a large sacrifice in explained variance (dropping a valuable atom): only if an algorithm knows it can immediately compensate by reintroducing the atom at a nearby time will this sacrifice be chosen by an optimization scheme.

We developed such an optimization scheme, by adding a step at every iteration where we allow existing spikes to change their location and/or magnitude to better account for the calcium trace. The optimal changes can be calculated exactly and efficiently at all small temporal offsets from existing atoms, by using the precomputed filtered trace. This step allows “old” spikes to adjust their locations and activity in the context of “new” spikes, thus reaching more accurate solutions.

We note that another algorithm for solving a similar problem has recently been proposed (Jewell and Witten, 2017). This algorithm does not impose positivity, is restricted to exponential kernels, and is slower than our approach, but it does obtain an exact solution for their respective problem, which our greedy approach does not.

##### Kernel choices.

For the NND, L0- and L1-regularized models, we used kernels that were either exponential or a difference of exponentials to model the fluorescence rise time. The timescale of the exponential was chosen as 1 of 3 possibilities: fast (0.7 s), medium (1.25 s), or slow (2 s), according to the calcium sensor used. Timescales for the GCaMP6 sensors were assigned according to their version. For other sensors, timescales were assigned based on the literature (OGB: medium; GCaMP 5k: fast; jRCaMP1a: slow; jRGECO1a: fast). To model fluorescence rise time, we used difference of exponentials, with the decaying exponential having the same timescale as before. The exponential accounting for the rising phase had a timescale that was varied as a fraction (0–0.5) of the timescale of the decaying exponential (see Figs. 2*e*,*f*, 3*h*).

##### Simulations.

To simulate fluorescence traces, we first generated the underlying spike trains. The number of spikes *s*(*t*) in bin *t* of 10 ms was simulated from a Poisson process, *s*(*t*) ∼ Poisson (*r*(*t*)), where the firing rate *r*(*t*) is constructed from the following:

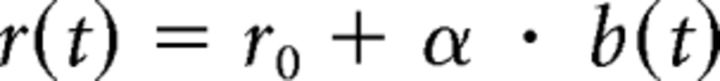
 Here, *r*_0_ is the baseline firing rate and α is a scaling factor that determines the ratio of bursting to tonic firing. *b*(*t*) is 1 during bursting and 0 otherwise. Bursting periods occurred randomly at least twice per minute and lasted for 250 ms.

We then simulated the calcium fluorescence by first filtering the spike train *s* with an exponential kernel (timescale 1 s) and adding independent Gaussian noise at each time point to simulate shot noise (see below). Finally, to simulate temporally correlated noise, such as movement and neuropil contamination, we added Gaussian-filtered white noise (half-width of 500 ms), to simulate other sources of noise, such as movement and neuropil contamination.

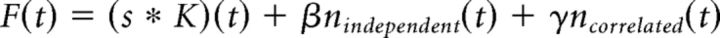
 The kernel was an exponential filter with a timescale of 1 s. To produce multiple datasets (see Fig. 2*g*,*h*), we randomly varied simulation parameters with a uniform distribution: mean firing rate (0.05–1 Hz), burstiness α (0–0.5), shot noise amplitude β (0–0.02), and correlated noise amplitude γ (0–0.015). The parameters were fixed within each simulated dataset of 200 cells.

In Figure 3*b*, we also use these simulations. For three fixed firing rates and with α = 0, we generated three sets of 200 spike trains. From each of these sets, we generated 20 datasets with different combinations of shot noise and correlated noise, but keeping the underlying spike trains fixed.

##### Performance metrics.

**Figure 1.**
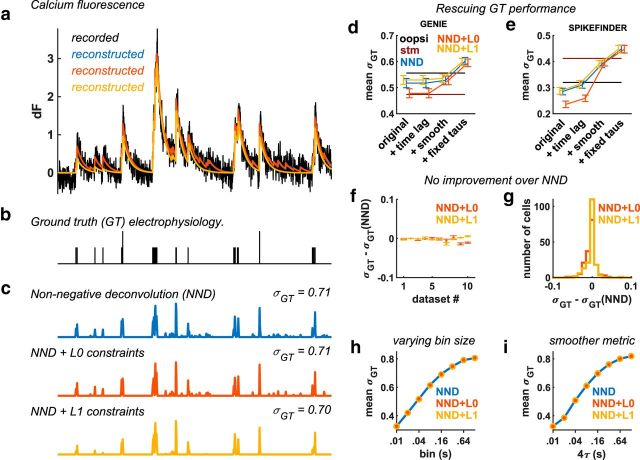
Deconvolution performance with ground-truth electrophysiology. ***a***, Example fluorescence recording of a neuron recorded by the GENIE Project. The model trace reconstructions are shown in color. Blue represents NND. Red represents NND + L0. Yellow represents NND + L1. ***b***, Simultaneous ground truth electrophysiology for the neuron shown in ***a***. ***c***, Deconvolved traces using three NND models. ***d***, ***e***, Correlation σ*_GT_* between deconvolved and ground truth spike trains, with various processing stages included, averaged over cells separately for datasets from the GENIE Project (2015) and for SPIKEFINDER datasets (Theis et al., 2016). The average values for stm and oopsi are taken from the spikefinder challenge (http://spikefinder.codeneuro.org/). ***f***, Per dataset, average improvement of L0- and L1-based methods over NND. ***g***, Distribution across cells of the improvement of L0- and L1-based methods over NND. ***h***, Mean σ*_GT_* across all datasets as a function of bin size. ***i***, Same as ***h***, but instead of binning, spike trains were smoothed with a Gaussian of standard deviation τ.

**Figure 2.**
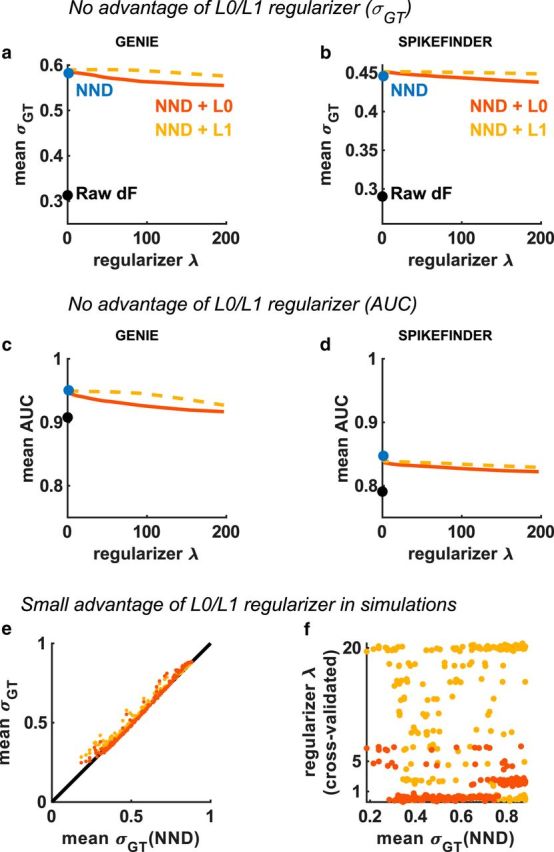
No performance advantage from L0/L1 regularization. Effect of regularization parameter λ on mean correlation σ*_GT_* or the AUC between deconvolved spike trains and ground truth electrophysiology. ***a***, ***b***, The penalty on sparsity was varied for both L0- and L1-penalized models. The best value of 0 corresponds to unconstrained NND. ***c***, ***d***, Same as ***a***, ***b*** for a different performance metric (area under the ROC curve, AUC). ***e***, ***f***, Performance of L0 and L1 methods versus NND, in simulations obtained by randomly varying 4 parameters: firing rate, burstiness, shot noise, and correlated noise. The optimal regularizer for L0 and L1 methods was chosen by cross-validation and is shown in ***f***.

**Figure 3.**
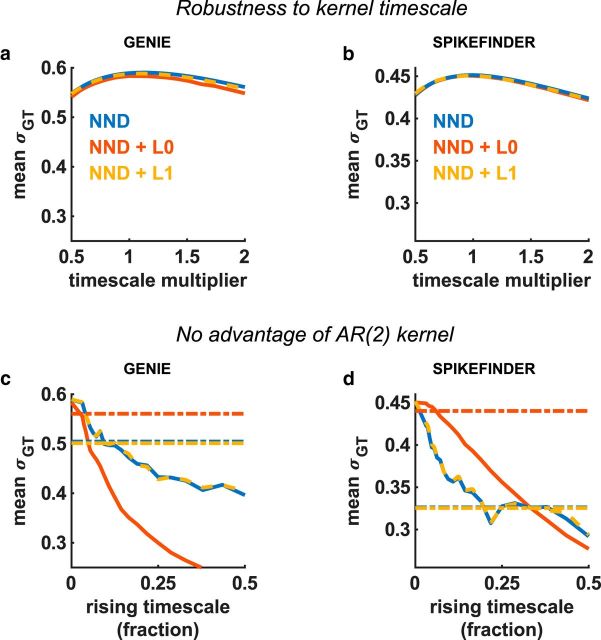
Robustness to kernel parameters. ***a***, ***b***, The kernel timescales were varied; this had little effect and did not significantly improve performance over values taken from the literature. ***c***, ***d***, A second kernel timescale was introduced and varied, to model the rise time of the fluorescence following a spike. The kernels were defined as a difference of exponentials, which defines a subclass of AR(2) kernels. The AR(2) version of OASIS was used for the L1-penalized method. Performance with ground truth-derived kernels is also shown as dotted lines (see Materials and Methods). The optimal rising timescale was 0, corresponding to a simple exponential kernel.

The main performance metric we used was σ*_GT_*: the Pearson correlation of a ground truth spike train **s***_GT_* and an estimated spike train **s**, both binned with 40 ms resolution (see Figs. 1, 2, 3). This was the same metric as used by the spikefinder challenge (Theis et al., 2016; Berens et al., 2018). We also used a variant of the van Rossum spike train distance, where both **s** and **s***_GT_* are smoothed with a Gaussian before taking their correlation (van Rossum, 2001; Schreiber et al., 2003). The width of the Gaussian was varied systematically (Fig. 1*i*).

We also used the area under the ROC curve (AUC) as a metric (Vogelstein et al., 2010; Theis et al., 2016) (Fig. 2*c*,*d*). The AUC can be computed as the fraction of times that the deconvolution result for a bin containing a spike is larger than the deconvolution result for a bin containing no spike. When both results are exactly equal to each other, we count this as a “half correct” value, or 0.5.

Finally, we introduce a new metric σ*_stim_* based on the stimulus response reliability. Specifically, σ*_stim_* is the Spearman correlation of trial-averaged tuning curves, obtained from two independent halves of a recording, where the stimuli were presented in random order on each half. Defining the trial-averaged responses of neuron *n* to stimulus *k* as *r*_1_(*k*, *n*) on the first repeat and *r*_2_(*k*, *n*) on the second repeat, the correlation σ*_stim_* of stimulus responses is simply as follows:

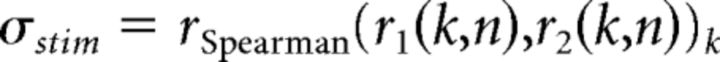
 where the *k* subscript indicates that the correlation is taken over stimuli *k*.

The correlation σ*_stim_* is not trivially 1 because the calcium signals are not identical across repeats. This trial-to-trial variability reflects (present tense) both genuine differences in neural spike trains, and the noisy transformation from electrical spiking to fluorescence. The task of a spike deconvolution algorithm is to invert this transformation, thereby denoising the recovered signals. The more successful an algorithm is, the more noise it will have removed, and the higher its σ*_stim_* value should be, even though these values cannot reach 1 due to neuronal variability.

We chose the rank correlation (Spearman) rather than the Pearson correlation to avoid potential biases introduced by nonlinearities in the spike deconvolution process. For example, if the result of deconvolution to both repeats was transformed through the same nonlinearity, the Pearson correlation could be artificially increased, whereas the Spearman correlation will remain the same.

##### CNNs.

Of the SPIKEFINDER challenge winners, one team has so far made their code publicly available (https://github.com/PTRRupprecht/Spikefinder-Elephant/tree/master/elephant), so we used their CNN configuration, called “Elephant.” This method fits >100,000 free variables, which were learned using default parameter settings.

## Results

For the first set of benchmarks, we considered two main classes of datasets with simultaneously recorded ground truth electrophysiology, which we will refer to in short as “GENIE” and “SPIKEFINDER.” The GENIE collection consists of five datasets recorded by the GENIE Project (Akerboom et al., 2012; Chen et al., 2013; GENIE Project, 2015; Dana et al., 2016), that have been used in the original descriptions of the GCaMP calcium sensors and their red variants and are available on www.CRCNS.org (GENIE Project, 2015). The SPIKEFINDER collection consists of the five datasets analyzed in Theis et al. (2016), made publicly available as part of the “spikefinder” challenge (http://spikefinder.codeneuro.org/). Surprisingly, the two state-of-the-art algorithms, “oopsi” and “stm” (Vogelstein et al., 2010; Theis et al., 2016), have different performance on the two GT datasets, with oopsi winning on GENIE and stm winning on SPIKEFINDER.

To measure deconvolution performance requires metrics to compare the algorithms' output to ground truth spike trains. A standard metric, termed σ*_GT_* throughout this paper, consists of the Pearson correlation coefficient of the true and inferred spike trains, after binning both in a preset window size (i.e., 40 ms in the spikefinder challenge, also the standard value used here). However, this metric may not be well suited for spike trains, which are very sparse quantities. For example, if an inferred spike is offset by just one bin from a GT spike, it will be counted as a complete miss by the correlation metric, similarly to a temporal mismatch of several bins. Supervised algorithms, if assessed on in-sample test data, will have automatic protection from this effect as they are directly trained to minimize the correlation metric; however, this behavior can lead to worse out-of-sample performance as shown below. Unsupervised algorithms can be adapted to perform well by the correlation metric by smoothing their output temporally, which reduces the effect of temporal mismatches between true spikes and deconvolved spikes. In addition, a temporal offset might be required for some datasets (e.g., due to hardware synchronization issues), which can be learned automatically by the supervised methods, but has to be inferred *post hoc* in the unsupervised algorithms.

A short segment of fluorescence from a cell recorded with ground truth is shown in Figure 1*a*, *b*, together with the reconstructions obtained with three unsupervised models (the quantity **s** * **k**; Eq. 1). All three models track the large fluorescence changes, but some of the smallest changes are only tracked by the unconstrained model (NND), which is relatively less constrained than the L0- or L1-penalized methods. For example, the L1-penalized model overly penalizes single spike events in some cases, reducing their amplitude relative to other single or multispike events (Fig. 1*c*). Nonetheless, for this cell, all three deconvolution methods returned approximately similar spike trains, which correlated well with the known ground truth electrophysiology (Fig. 1*b*,*c*).

### Simple NND outperforms the state-of-the-art results

Many calcium deconvolution algorithms have recently been described (Vogelstein et al., 2010; Andilla and Hamprecht, 2014; Reynolds et al., 2015; Deneux et al., 2016; Theis et al., 2016; Friedrich et al., 2017; Jewell and Witten, 2017; Sebastian et al., 2017; Kazemipour et al., 2018), some of which have provided their code publicly. However, little effort has been made to compare the performance of these algorithms with each other, with the notable exception of Theis et al. (2016) who concluded that supervised algorithms, trained on available ground truth data, perform better than the more routinely used unsupervised algorithms.

We investigated this claim on the same datasets used by Theis et al. (2016), which were since made available publicly in the “spikefinder” challenge. We indeed found that the supervised algorithms performed better than the L0- and L1-penalized algorithms, when evaluated by the correlation metric (Fig. 1*d*,*e*, “original”). However, we found that unsupervised algorithms became superior after very simple modifications.

First, we chose an appropriate timelag parameter for a subset of datasets where the timing of the ground truth spikes was not perfectly synchronized with the fluorescence (Fig. 1*d*,*e*, “+time lag”). This parameter was chosen to maximize the correlation with the ground truth spikes, for each of the 10 datasets separately. The inferred timelag was 0 for all GENIE datasets and ranged between −2 and 3 for SPIKEFINDER datasets.

Second, we applied smoothing to the deconvolved traces (but not to the ground truth spike trains), which reduced the effect of the correlation metric (Fig. 1*d*,*e*, “+smoothing”). The smoothing was performed with a Gaussian-shaped kernel with an SD of two samples for all GENIE datasets, and 8 samples for all SPIKEFINDER datasets. (These values were empirically found to perform well.) All datasets have been upsampled at 100 Hz and are benchmarked at 25 Hz, following the standards of the original spikefinder benchmarks.

Finally, we did not allow the algorithms to estimate the best fit calcium kernels, or the kernel's decay timescale, as we found that all methods failed to recover appropriate parameters. Instead, we fixed the timescales of the calcium kernel to be approximately the measured values from the literature (Akerboom et al., 2012; Chen et al., 2013; Dana et al., 2016) (Fig. 1*d*,*e*, “+fixed taus”). For simplicity, we divided all sensors into a fast, a medium and a slow category, assigning them corresponding timescales of 0.75, 1.25, and 2 s. As we show below, the precise values for these timescales were not critical.

These improvements, together, increased the benchmark performance for nearly all cells, and surpassed both the supervised and unsupervised “state-of-the-art” approaches submitted on the website spikefinder by their developers (stm and oopsi). Furthermore, the best performing model in the benchmark was unconstrained NND, with the L0- and L1-based methods slightly lagging behind (Fig. 1*d*,*e*). Across datasets, unconstrained NND reliably performed as well as or better than L0- and L1-based methods (Fig. 1*f*), with only small differences between models. Similarly, the differences were small at the level of single cells (Fig. 1*g*), suggesting that it was not the case that some cells were better deconvolved by some models.

To test the performance of the algorithms at different timescales, we varied the binning size from 10 ms to 1.28 s. Again, we found the three top algorithms to be virtually indistinguishable (Fig. 1*h*). To test whether the distance metric used to evaluate spike train accuracy might influence the result, we turned to a variant of the van Rossum distance (van Rossum, 2001; Schreiber et al., 2003), which smoothed the spike trains before computing correlations. Over a wide range of smoothing widths, the correlation between ground truth and estimated spike trains followed a similar pattern, with all three unsupervised algorithms performing very similarly (Fig. 1*i*).

### Robustness of NND

These results suggest that the choice of regularization method (if any) does not have a major impact on the performance of unsupervised deconvolution. We tested this more systematically, by varying the regularization parameter λ. When λ = 0, both the L0- and L1-based algorithms are equivalent to the unconstrained approach. We found that, for both algorithms, performance was best when λ = 0, corresponding to unconstrained NND (Fig. 2*a*,*b*). We confirmed this result with another metric: the area under the ROC curve (AUC) (Fig. 2*c*,*d*), which can be computed as the fraction of times that the deconvolution result for a bin containing a spike is larger than the deconvolution result for a bin containing no spike.

These results suggest that the simple NND method consistently matches or outperforms L0 and L1 methods. However, the GENIE and SPIKEFINDER datasets contain several tens of cells each, which may not be a large enough sample to test how generally this result holds. To extend the range of our analysis, we generated simulated datasets where we varied for each neuron its firing rate, burstiness, shot noise amplitude, and correlated noise amplitude. Each simulated dataset was generated under a random combination of these four parameters. For this analysis, we chose the value of the regularization parameter for L0 and L1 methods that gave optimal test set performance: these methods can therefore choose a value of 0 if that performs best, and thus by definition cannot perform worse than simple NND. Nevertheless, we observed only minimal improvements due to regularization, and only in a small number of scenarios (Fig. 2*g*). The regularizer was most often set to near zero, but sometimes high values were chosen by the L1-based method (Fig. 2*f*). We conclude that regularization provides little, if any, benefit in both simulated data and real data with available ground truth.

The unsupervised algorithms were robust not just to the value of the regularizer, but also to large changes in the shapes of the calcium kernels. Lengthening or shortening the assumed timescale of the calcium indicators by a factor of 2 did not significantly affect performance (Fig. 3*a*,*b*). Furthermore, adding another component to the kernel (a “rising” timescale) did not improve performance (Fig. 3*c*,*d*). Finally, performance was not improved by using the “ground truth” kernel, obtained directly by regressing the fluorescence onto the ground truth spikes (Fig. 3*c*,*d*, dotted lines).

### Benchmarks without ground truth using stimulus responses

**Figure 4.**
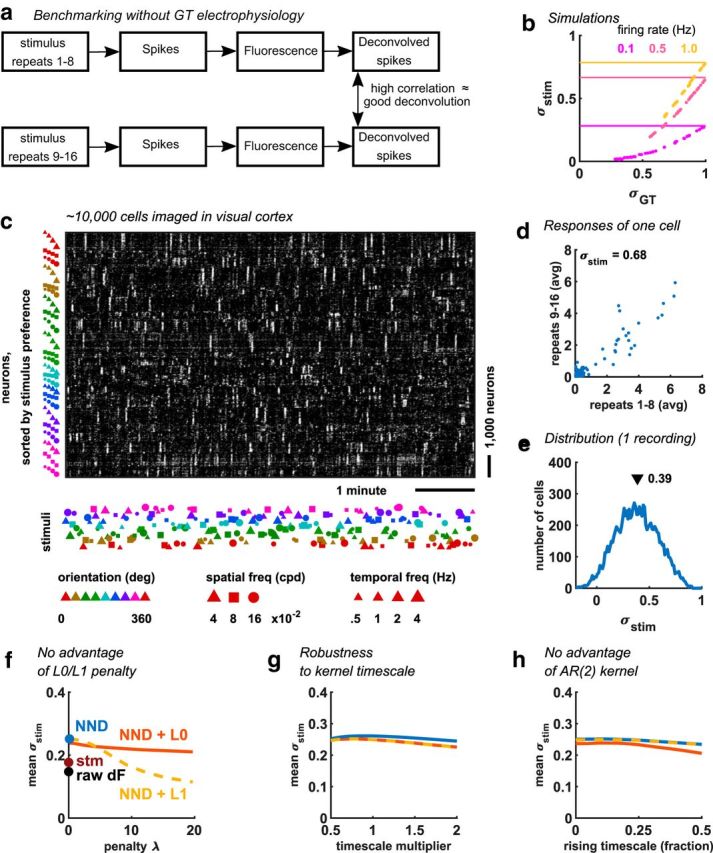
Benchmarking without ground truth electrophysiology. ***a***, Schematic of the processes leading up to the deconvolved spike trains. ***b***, The new performance metric σ*_stim_* plotted against (σ*_GT_*), for three example simulated spike trains. Dots of a single color indicate different instantiations of the same spike train, with different added noise and different deconvolution algorithms. Horizontal lines indicate the maximum possible. ***c***, Example NND (algorithm) from ∼10,000 simultaneously recorded neurons, sorted by their preferred stimulus. The stimuli shown were drifting gratings with 1 of 8 directions, 1 of 3 spatial frequencies, and 1 of 4 temporal frequencies. ***d***, Correlation between trial-averaged neural responses to half of the stimulus presentations versus the other half of the stimulus presentations. ***e***, Distribution of correlation coefficients between repeat halves (Spearman). The mean of these coefficients is used in ***f–h*** as a benchmark of deconvolution performance (higher is better). ***f***, Deconvolution performance as the penalty on sparseness was increased, for L0- and L1-penalized models, as well as for raw nondeconvolved data, and for the stm model from Theis et al. (2016). ***g***, Deconvolution performance as the timescales are increased or decreased by a fractional amount. ***h***, Performance with AR(2) kernels that include a noninstantaneous rise with varying timescales.

NND therefore exceeds the performance of supervised methods, and at least matches the performance of L0- and L1-regularized based methods in both the SPIKEFINDER and GENIE datasets, as well as a range of simulations. Still, however, these conditions might be different from the conditions in many experiments. For example, the SPIKEFINDER and GENIE datasets were recorded under anesthesia, with an invasive electrode attached to the cell. To assess the performance of spike deconvolution methods in realistic recordings, we developed a novel benchmark that does not require ground truth electrophysiology (Fig. 4*a*).

Our new benchmark is based on the intuition that a more accurate spike detection method will yield more similar responses to repeated stimuli. Specifically, this benchmark is the Spearman correlation σ*_stim_* of deconvolved responses, trial-averaged on two separate halves of the data: σ*_stim_* = corr(*ŝ*_*repeat*1_, *ŝ*_*repeat*2_), where *ŝ*_*repeat*1_ and *ŝ*_*repeat*2_ are the binned, trial-averaged responses to *N* different stimuli. The stimuli must be presented in randomized order on each of the two repeats, so that the only common information contained in the neural responses is related to the spiking activity. σ*_stim_* computes a trade-off between signal and noise variance, with 1 − σ*_stim_* representing the proportion of noise variance (reflecting a sum of biological and measurement noise; see Materials and Methods). Because the signal can only originate in the true spiking **s**, σ*_stim_* captures the ability of the deconvolution to reconstruct the true spiking. Deconvolution can fail to capture the stimulus variance in **s** in one of two ways: (1) failure to distinguish spikes from noise or (2) failure to correctly invert the forward calcium model, for example assigning spikes to incorrect stimulus bins. Even with perfect spike detection, the actual spike train would also differ between repeats; thus, σ*_stim_* cannot reach 1 but will have a maximal value, given by the Spearman correlation of the underlying spike trains. This maximum will be achieved when the deconvolved spike trains exactly match the original spike trains at the time resolution used to quantify stimulus responses.

This new metric gave nearly identical results to the standard metric on simulated data. To show this, we first generated three sets of spike trains of different mean firing rates, in response to multiple repeats of simulated stimuli. From each set of spike trains, we generated multiple fluorescence traces by varying the noise parameters of each simulation (shot noise and correlated noise) and then ran multiple deconvolution algorithms on each trace, using multiple parameter settings. For each spike train, the deconvolution performance as assessed by σ*_stim_* was nearly perfectly correlated with performance assessed by the standard measure σ*_GT_* (the correlation between deconvolved spike trains and the true underlying spike trains) (Fig. 4*b*). Nevertheless, this relation could not be extrapolated between spike trains: for the same value of σ*_GT_*, different spike trains could have different values of σ*_stim_*. Thus, σ*_stim_* can be used to compare the relative deconvolution performance of multiple algorithms on the same spike train, but not to estimate on an absolute scale how similar the deconvolved spikes are to the true, unknown spikes.

Having established σ*_stim_* as a useful metric for comparing algorithms, we applied it to some 2-photon recordings of ∼10,000 cells from primary visual cortex of awake mice (Stringer et al., 2018), with full field drifting grating stimuli of varying orientation, spatial, and temporal frequency. A raster plot of these responses (deconvolved by NND) is shown in Figure 4*c*. After averaging over 8 repeats, the responses of many single cells were generally reliable but still contained some noise (Fig. 4*d*). Nearly all cells had positive Spearman correlations σ*_stim_* of their tuning curves between the two stimulus halves (Fig. 4*e*). Taking σ*_stim_* as a measure of deconvolution performance (higher is better), we repeated the types of analyses from Figures 2 and 3, for 6 datasets recorded from 4 mice. Again, we found that simple NND outperformed the regularized versions, as well as the supervised stm algorithm, which in turn only slightly outperformed the raw fluorescence (Fig. 4*f*). We also again found that the NND methods (with or without regularization) are robust to the kernel timescale up to a factor of 2 (Fig. 4*g*), and that the AR(2) kernel does not help performance (Fig. 4*h*). This new metric therefore reinforces the results obtained on the more limited dataset with simultaneous electrophysiology. In addition, we note that this method can be easily applied to other recordings, with as few as two repeats of the same stimulus, allowing users to benchmark multiple approaches on their own data. We provide a code repository and example data to facilitate these comparisons (Pachitariu et al., 2018) (code: https://github.com/MouseLand/pachitariu-et-al-2018a).

### Comparisons with CNNs

We have shown that unsupervised methods, when adapted to the correlation-based benchmark, outperform the supervised approach described by Theis et al., (2016), on the same ground truth data used there. However, it remains possible that other supervised methods might perform better still. In the recent “spikefinder” challenge, CNNs appear to outperform all other methods. However, to successfully apply CNNs on standard recordings without ground truth, it must be shown that CNNs generalize to datasets not included in the training set. We suspected this generalization might be imperfect because CNNs, like all supervised methods, can overfit to the specific statistics of the ground truth datasets.

**Figure 5.**
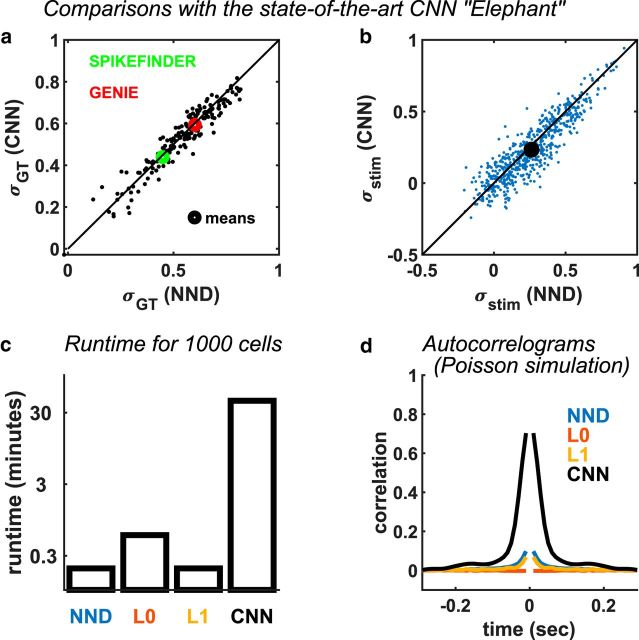
NND matches the performance of supervised CNNs. ***a***, Spike detection performance (σ*_GT_*) for all cells with ground truth electrophysiology. The means for all GENIE and Theis et al. (2016) datasets are shown as circles. Each CNN was trained on all but one of the 10 datasets and tested on the remaining dataset. ***b***, Stimulus response correlation (σ*_stim_*) of CNN trained on all 10 datasets and tested on our data using the repeat similarity benchmark (Fig. 3). ***c***, Runtimes of CNN on a high-end GPU (GTX 1080), compared with runtimes for NND implemented by OASIS (with or without L1 regularization) and the L0 method on a standard CPU (Core i7). ***d***, Auto-correlograms of deconvolved spike trains from simulations with Poisson ground truth statistics. The CNN approach heavily biases the statistics of the inferred spike trains. The L0 and L1 methods were run with same parameters as in Figure 2 (λ = 10 and λ = 100, respectively).

To evaluate the generalization performance of CNNs, we trained the “Elephant” method on 9 of the 10 available datasets, testing it on the 10th. We found that, under this testing protocol, the performance of supervised CNNs and unsupervised NND was nearly identical, both on the GENIE datasets (CNN: 0.44; NND: 0.45) and on the SPIKEFINDER datasets (CNN: 0.59; NND: 0.60), although there were slight variations in performance from neuron to neuron (Fig. 5*a*). However, the performance of the CNNs was worse in the ground-truth free benchmarks we just introduced above (CNN: 0.234 vs NND: 0.262), with some variation from neuron to neuron (Fig. 5*b*).

Another disadvantage of complex CNNs is speed: even using a high-performance GPU (GTX 1080), the method is two orders of magnitude slower than all unsupervised methods we tested, which can run efficiently on standard CPUs (Fig. 5*c*).

### Supervised methods impose biases on out-of-sample data

The similarity in performance we found might appear at odds with the results of the spikefinder challenge, where the CNN methods outperformed unsupervised approaches by ∼10% (CNN: ∼0.46 vs NND+L0: ∼0.43). However, for that challenge, the CNN was tested on within-class data, thus being able to take advantage of the particular statistics of spiking and fluorescence for each recording. One such statistic is the auto-correlogram structure of the spike trains, which was far from Poisson, reflecting either that stimuli were presented during some of the recordings or the structure of spontaneous activity in the recorded neurons. The auto-correlogram structure can be used by supervised approaches to perform better spike prediction. However, this strategy is undesirable because it will enforce the properties of the training data on new data, potentially leading to an erroneous scientific conclusion that all recorded neurons share the same temporal dynamics as the neurons used to train the algorithm.

To demonstrate this transfer of constraints between training and test data, we simulated spike trains with Poisson statistics (flat auto-correlograms) and generated fluorescence traces from them with a calcium decay timescale of 1 s. The deconvolved spike trains using CNNs had a large, spurious auto-correlation at short timelags, which was much less pronounced in methods based on OASIS (NND and NND+L1) and absent using the L0-based method (Fig. 5*d*). The “black-box” nature of CNN algorithms raises a further concern that other features of the training data may be erroneously imposed on new data, in ways that are unknown to the user.

## Discussion

We conclude that the performance of simple NND-based deconvolution algorithms matches or exceeds all tested alternatives. Adding L0/L1 regularization to the NND model did not improve its performance, perhaps because non-negativity is already a strong regularizer by itself. NND was robust to changes in kernel timescale and shape, with values taken from the literature providing close to optimal performance. Automated identification of kernel parameters appeared to be counterproductive, resulting in mismatched parameters that impaired performance. While supervised methods gave apparently superior performance in previously reported benchmarks, this reflected their ability to optimize particular evaluation metrics and compensate for phenomena such as synchronization lags within single example datasets. When tested out-of-sample, against unsupervised methods with appropriate compensatory mechanisms, we found their performance to be inferior.

The potential pitfalls of using more complex methods go beyond poor out-of-sample generalization. These methods may also introduce biases, usually due to their implicit or explicit priors. For example, L0- and L1-based deconvolution may introduce too much sparsity into the spike trains. Supervised models do not typically impose explicit priors but learn implicit priors from the statistics of the training data. They then impose these priors on new test data, as we have shown here (Fig. 5*d*), even if the new data have different statistics, potentially leading to erroneous scientific conclusions.

We therefore recommend simple, unconstrained NND, with fixed calcium decay timescale. OASIS (Friedrich et al., 2017) provides a very efficient algorithm for performing this deconvolution. In Suite2p, the calcium processing pipeline that we maintain, we provide wrappers for the OASIS toolbox, and additionally include the L0-based deconvolution code, which may provide advantages for some cases, such as avoidance of autocorrelation bias (Fig. 5*c*). It remains possible that future methods will be able to significantly outperform simple NND; however, any such improvements need to be balanced against the simplicity and interpretability of the NND approach.
